# Activity painting: PET images of freely defined activity distributions applying a novel phantom technique

**DOI:** 10.1371/journal.pone.0207658

**Published:** 2019-01-25

**Authors:** Attila Forgacs, Piroska Kallos-Balogh, Ferenc Nagy, Aron K. Krizsan, Ildiko Garai, Lajos Tron, Magnus Dahlbom, Laszlo Balkay

**Affiliations:** 1 Scanomed Nuclear Medicine Center, Debrecen, Hungary; 2 Division of Nuclear Medicine, Department of Medical Imaging, Faculty of Medicine, University of Debrecen, Debrecen, Hungary; 3 Ahmanson Translational Imaging Division, University of California at Los Angeles, United States of America; US Department of Agriculture, UNITED STATES

## Abstract

The aim of this work was to develop a novel phantom that supports the construction of highly reproducible phantoms with arbitrary activity distributions for PET imaging. It could offer a methodology for answering questions related to texture measurements in PET imaging. The basic idea is to move a point source on a 3-D trajectory in the field of view, while continuously acquiring data. The reconstruction results in a 3-D activity concentration map according to the pathway of the point source. A ^22^Na calibration point source was attached to a high precision robotic arm system, where the 3-D movement was software controlled. 3-D activity distributions of a homogeneous cube, a sphere, a spherical shell and a heart shape were simulated. These distributions were used to measure uniformity and to characterize reproducibility. Two potential applications using the lesion simulation method are presented: evaluation in changes of textural properties related to the position in the PET field of view; scanner comparison based on visual and quantitative evaluation of texture features. A lesion with volume of 50x50x50 mm^3^ can be simulated during approximately 1 hour. The reproducibility of the movement was found to be >99%. The coefficients of variation of the voxels within a simulated homogeneous cube was 2.34%. Based on 5 consecutive and independent measurements of a 36 mm diameter hot sphere, the coefficient of variation of the mean activity concentration was 0.68%. We obtained up to 18% differences within the values of investigated textural indexes, when measuring a lesion in different radial positions of the PET field of view. In comparison of two different human PET scanners the percentage differences between heterogeneity parameters were in the range of 5–55%. After harmonizing the voxel sizes this range reduced to 2–16%. The general activity distributions provided by the two different vendor show high similarity visually. For the demonstration of the flexibility of this method, the same pattern was also simulated on a small animal PET scanner giving similar results, both quantitatively and visually. 3-D motion of a point source in the PET field of view is capable to create an irregular shaped activity distribution with high reproducibility.

## Introduction

Positron emission tomography (PET) integrated with computer tomography (CT) is capable to quantitatively measure the distribution of a radioactive tracer with positron decay in the human body. The measured distribution of radioactive nuclides can reflect biological properties due to the role of the labelled molecule. Therefore, PET is a non-invasive diagnostic tool assessing staging, treatment selection, and patient follow-up. The possibility to extract information from the reconstructed PET images is an inevitable trend and challenge, leading to personalized medicine. According to the aspiration of radiomics [1–5], not only the presence at a certain location, volume and standardized uptake value (SUV) of a lesion can hold diagnostic value, but also its texture and shape. The apparent texture of a lesion on the reconstructed image is strongly but indefinably affected by noise, partial volume effect (PVE), and reconstruction parameter settings [6–12]. On the other hand, the number of possible parameters to measure textural properties of a lesion are practically unlimited [13], thus the significance of potential candidates needs to be proven in a validation process [9,14]. Conventional methods use various types of phantoms with known geometry and activity concentrations. Very few of them have been constructed primarily to answer methodologic questions related to the measurement of texture [15]. Plastic phantoms equipped with fillable compartments, such as the NEMA IEC body phantom and the Jaszczak phantom, are commonly used in PET [16]. The structures within these phantoms are typically limited to relatively simple geometrical objects (spheres, rods, cylinders) which allows straightforward and reproducible preparation. However, the results from these measurements are not easily translated to express the system’s ability to measure texture in a realistic situation, since the compartments are limited to be filled with uniform radioactivity distributions. In a previous study, our group proposed a so called “revolver phantom” consisting of seven 2 ml syringes to mimic a heterogeneous activity uptake [9]. This phantom preparation was more complex, but still utilizing a relatively simple geometry. A heterogeneous activity distribution can be produced using phantom structures made from zeolite [17], however, the irregular activity distribution inside the zeolite cannot be controlled [18]. Another phantom type is the 2-D printed phantom [19,20], where the radioactive isotope is mixed with the printer ink. The method requires a careful calibration of the grey scale image the activity concentration of the image that is actually printed. The methodology can be extended to produce a volumetric phantom where multiple layers of 2-D printed sheets are stacked together to form a 3-D volume [21]. This method can therefore be very time consuming (comparable to the half-life of ^18^F) if large and complex distributions are to be produced. 3-D printing technology can also be used to manufacture phantoms by incorporating the radioactive tracer in the cellulose powder used in some rapid prototyping system [22]. The short half-life of the most common PET isotope (^18^F) makes it difficult to perform comparative studies without requiring re-printing of the phantom.

Carles et al. [23] reported on a heterogeneous phantom study dealing with the evaluation of the complementarity of textural features and how feature values are effected by motion and the segmentation method. The heterogeneous ^18^F-FDG distribution phantom in this study was made from alginate. A comparison of PET images obtained at multiple research sites (multi-center study) would require to reproduce this phantom at each site, raising the question of reproducibility. Although the coefficient of variance (COV) was > 0.3, the level of heterogeneity for the evaluation textual features was determined primarily by the variance of voxel values, however, the heterogeneity of spatial pattern was not controlled. In contrast, PET raw data manipulation is an elegant, but complex way to generate arbitrary activity distributions [24]. It assumes the scanner list mode data structure and the scanner geometry is known, which is not always the case. Similarly, if the scanner geometry is known with high accuracy and mathematical anthropomorphic models are available, the Geant4 application for tomography (GATE) Monte Carlo simulation toolkit provide the possibility to generate realistic patient images, including heterogeneous tumors [25]. However, including detailed modeling of key components of a PET system (basic scanner geometry, scintillator material and the related block structure, signal generation on the used photodetector and the applied signal processing) results in long simulation times. Furthermore, to generate the most realistic synthetic data, simulation of the scintillation light photon transport is essential, may result in computational times that are excessively long. In addition, the raw data should be reconstructed with the vendor specific algorithm. Although the generation of synthetic PET data is beneficial to an improved understanding of the nature of textural characterization [26,27], simulations cannot replace measurements on actual imaging systems.

The variability of radiomics features, due to the different scanner model, acquisition protocols or reconstruction settings can hide the biological effect [12,28,29], especially in case of inter-device or multicenter studies. Our aim was to develop a method to measure the inter-scanner variability of textural feature measurements with high accuracy while facilitating comparisons of phantom studies across multiple sites. In this work we present a method to create irregular 3-D activity distributions with high reproducibility based on a controlled movement of a point source in the PET field of View (FOV).

## Materials and methods

### Source positioning system

**Fig 1:**
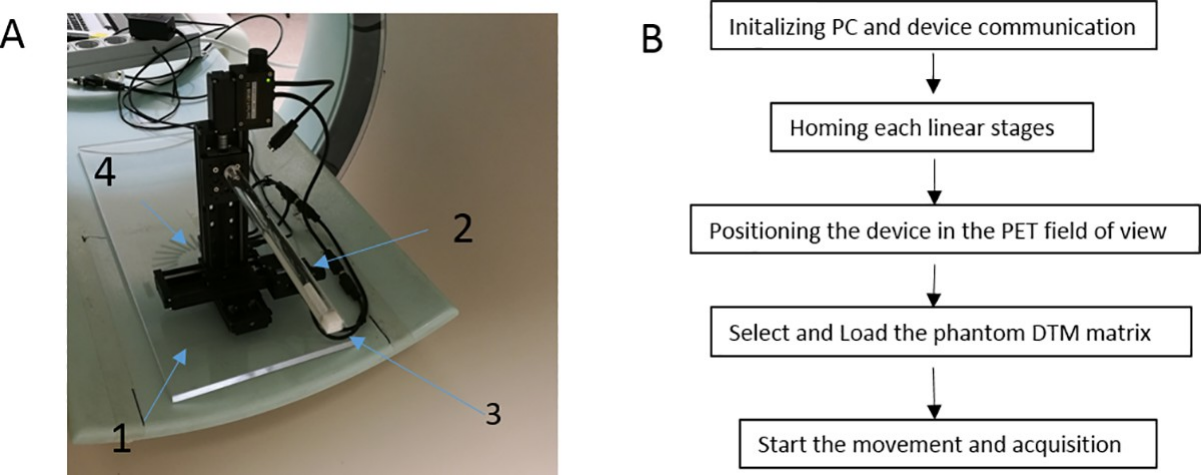
Positioning system (A) and the flow diagram of the measurement procedure (B) The positioning system is consist of **a** plastic holder (arrow 1), plastic rod (arrow 2), ^22^Na point source (arrow 3), and linear stages (arrow 4).

Three linear stages (Zaber Technologies, T-LSM050A motorized stage, Fig 1) were mounted on a plastic plane holder (arrow 1) and placed on the patient bed. Three linear actuators (arrow 4) allow motion along the x, y and z axis independently. A 1.1MBq ^22^Na calibration point source (Eckert Ziegler) (arrow 3) designed for National Electrical Manufacturers Association (NEMA) performance tests was attached to the plastic rod (arrow 2) and moved within a volume of the PET FOV with a given step size in each direction. The motion was controlled by an in-house developed MatLab R2016b program. The speed of each linear stage was set to a common value in order to minimize artefacts due to the differences in positioning time. The assembled device and flow diagram of the measurement procedure are shown in Fig 1.

The point source was moved on a continuous pathway along a 3 dimensional cubic grid, and stopped in each of the grid points. According to the expected phantom image, a 3 dimensional time matrix assigns a stoppage or dwell time for each of the grid points (dwell time matrix DTM). The relative dwell times during the scan are converted to relative activity concentrations on the reconstructed image. The values of DTM was defined either mathematically (spheres, spherical shell), created manually (heart shape), or extracted from real reconstructed PET images. Different DTM’s were created and stored in a separate library. From the aspect of the measurement procedure (e.g. to mimic any irregular or regular images) just the total acquisition time will differ according to the imported DTM, whereas all other parameters were left constant (e.g., step size, speed of the movement). The step size of the device was optimized based on a set of acquisitions performed on the AnyScan PET/CT. During the experiment the point source was moved at a given transaxial plane along different lines. The step length varied from 4 to 10 mm and the dwell time was kept constant at 1 sec for each position.

### Scanners

Two clinical PET/CT systems were used in this work: A Disovery MI (GE Healthcare) equipped with digital Silicon Photomultiplier (SiPM) detectors and an AnyScan PET/CT (Mediso Ltd.) with conventional Photomultiplier Tubes (PMTs). In addition, a nanoScan PET/MRI (Mediso Ltd.) small animal scanner was also used in this study. Performance parameters of the systems relevant to this study are detailed in Table 1 [30,31].

**Table 1:** Technical parameters and reconstruction settings.

Scanner	Mediso AnyScan PET/CT	Mediso nanoScan PET/MRI	GE Discovery MI
**default voxel size [mm]**	4	0.4	2.73
**reconstruction iteration**	6	8	3
**reconstruction subset**	6	4	8
**Point Spread Function (PSF) correction**	+/-	+	+
**axial spatial resolution FWHM** **at a given radial position**	4.58mm^a^ rad. pos. 10mm	0.91mm rad. pos. 5mm	4.57mm rad. pos. 10mm
**Time of flight**	-	-	+

+ and—denotes the correction on or off

^a^measured by the authors

Images were reconstructed without scatter and attenuation correction since all measurements were performed with the point source in the air. The approximately 10% attenuation of the 1cm^3^ cube was neglected.

### Radiomics feature extraction

All image analysis was performed with the InterView Fusion Software (Mediso Ltd). The reconstructed lesions were delineated using a 40% threshold of the maximum voxel value. No post reconstruction processing was applied such as interpolation, filtering or other corrections. First order statistics, like standard deviation, mean, max values, coefficient of variation (COV) were calculated as well as higher order parameters based on grey level co-occurrence matrix (Contrast, Correlation, Entropy, Homogeneity), grey level size-zone matrix (Low Grey-Level Zone Emphasis (LGZE) and High Grey-level Zone Emphasis (HGZE)) and grey level run length matrix (Short-Run Emphasis (SRE), Long-Run Emphasis (LRE) and Run Length Non-Uniformity (RLNU)). The volumetric analyses were performed as a fully-connected 3-D volume, applying 64 bins discretization method [32]. In the case of the co-occurrence matrix, only the closest neighbors were considered. The feature calculations match the benchmarks of the Image Biomarker Standardization Initiative (IBSI) [13], with the exception of two terminologies (Entropy called Joint Entropy, homogeneity called Inverse Difference by IBSI).

### Patient data

In addition to the phantom simulation, lesion image data were extracted from a human study. The subject was included in the study reported in Ref [9]. This previous study has been approved by the local ethics committee. The images of the human data were anonymized prior to given to the authors of this study.

### Reproducibility

To measure the reproducibility, 5 consecutive scans were acquired on the Mediso AnyScan PET/CT. During each scan a hot sphere with 36mm diameter was simulated, positioned at the center of a 48x48x48mm^3^ cube. The ratio of activity concentration between the hot sphere and residual volume of the cube (background) was kept 3:1. Quantification of the reproducibility is expressed as the coefficient of variation of the mean, max, min values both for the background and for the hot sphere. The coefficient of variation was also calculated for the acquisition times since the variance of the total time reflects the uncertainties in the movements of the positioning system.

### Position dependency

Sampling frequency and spatial resolution of PET are not uniform across the field of view (FOV). The textural distortion resulting from the positional dependency in the FOV was also examined. An simulated 3-D heterogeneous lesion (size of 48x48x48 mm^3^) extracted from a human image was measured at 4 different radial positions (0, 30, 50 and 100 mm off from the center of FOV) on the Mediso AnyScan clinical PET/CT. The textural parameters and coefficient of variation were calculated after delineation of the simulated lesion by an isocontour method. Both PSF corrected and non-corrected reconstructions were performed and evaluated.

### Scanner comparison

Textural characterization and scanner comparison were evaluated based on the measurement of the same irregular texture activity distribution on Mediso AnyScan and GE Discovery MI. Isocontour delineation was followed by the calculation of the textural parameters and coefficient of variation. Visual interpretation and quantitative comparison in terms of Entropy, Homogeneity, Contrast, Correlation, COV were also performed [9].

### Scale independence

To demonstrate the flexibility of the method a heterogeneous lesion was also simulated in a nanoScan PET/MRI small animal system. In this case, the step size was reduced from 4mm to 1mm according to the difference in spatial resolutions (Table 1), the DTM matrix was kept identical to the clinical scanner measurements.

## Results

### Proof of the concept

**Fig 2:**
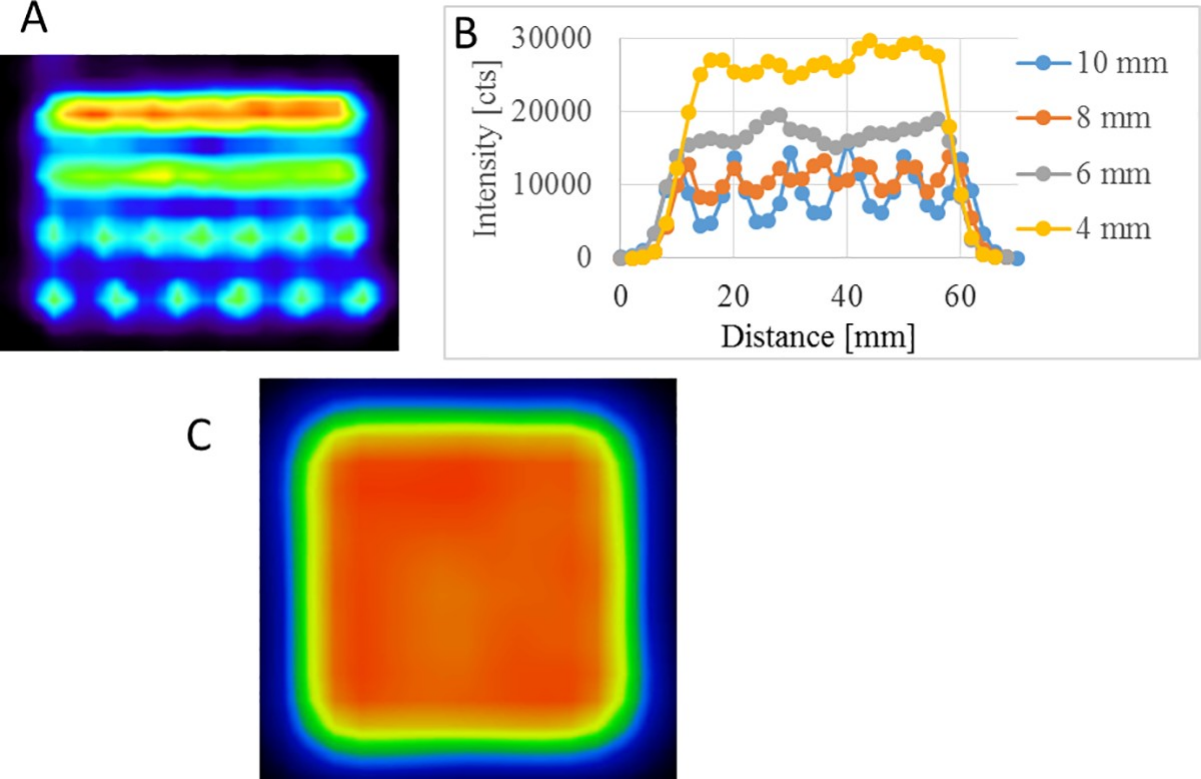
Step size optimization results. Lines having various step size (4, 6, 8, 10 mm) of the point source (A), derived line profiles (B) and transaxial slice of a 3-D homogeneous cube using 4mm step size between the point source movements (C).

The step size optimization (varied from 4 to 10 mm, constant 1 sec dwell time) resulted in the reconstructed transaxial image displayed on Fig 2A. Each line corresponds to a cross-section of a plane with a given step size (A). The line created by the 4 mm step size resulted an intensity profile of acceptable uniformity (Fig 2B). A smaller step size could improve the uniformity, but at the cost of scanning smaller volume or extended scan time. Panel C shows a transaxial slice of a 3-D uniform cube (48x48x48mm) utilizing the selected 4mm step size. Placing a 20 ml spherical volume VOI within the center of the cube resulted in a 2.34% of COV of the voxel values. This value is significantly lower than the uniformity value obtained with a standard IQ phantom when measured according to the European Association of Nuclear Medicine FDG PET/CT accreditation (EARL) guideline [33].

**Fig 3:**
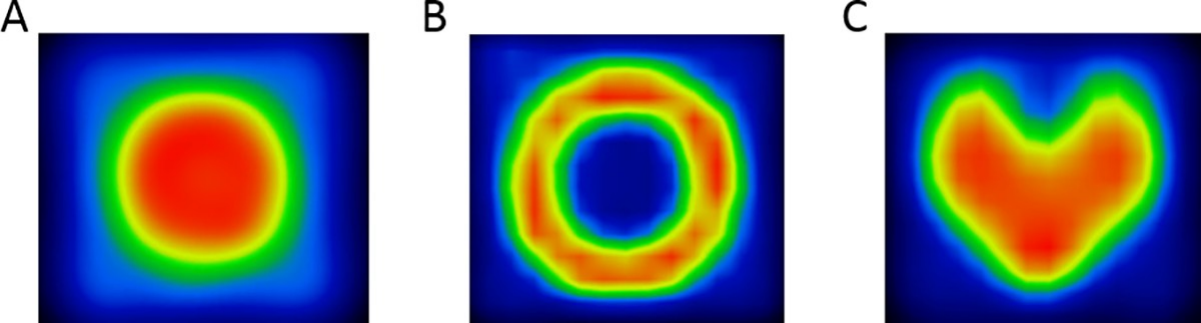
**Demonstrative axial slices of 3 dimensional images**: hot sphere (A), spherical shell (B), heart (C).

Based on the results shown on Fig 2, the 4 mm step size was selected. Different geometrical objects were simulated with the positioning system. A sphere, a spherical shell, and a heart shaped 3-D activity distributions were defined with the same dwell time (1 sec at inside the given shape and zero sec out of it) but different positioning maps in the same volume (48x48x48mm^3^ cube). Representative images are shown in Fig 3.

**Fig 4:**
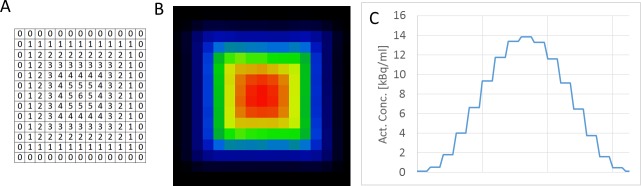
Activity concentration as the function of dwell time. The dwell time matrix in sec (A), the corresponding activity distribution on the reconstructed PET image (B) and the line profile through the center of the image (C).

Fig 4A shows the DTM for a cube where the dwell time was linearly increased from the surface to the center. Fig 4B shows the corresponding reconstructed image and a line profile through the image (Fig 4C). We found that the dwell time of the point source from 0 to 6 seconds generated 0 to 14 kBq/ml activity concentrations for the AnyScan PET/CT.

### Reproducibility

The values of the coefficient of variation of mean, max, min value of 5 consecutive measurements of a 36 mm diameter sphere are shown in Table 2. In addition, the COV of the time required to complete the source positioning was also measured as a measure of movement precision.

**Table 2:** Coefficient of variation of mean, max, min values and the total positioning time of 5 independent measurement of the phantom imitating 36 mm diameter sphere.

Coefficient of Variation [%] of						
Mean_hot_	Mean_bg_	Max_hot_	Max_bg_	Min_hot_	Min_bg_	Total positioning time
0.68	3.65	1.57	2.23	1.52	3.65	0.038

### Application 1. Position dependence

**Fig 5:**
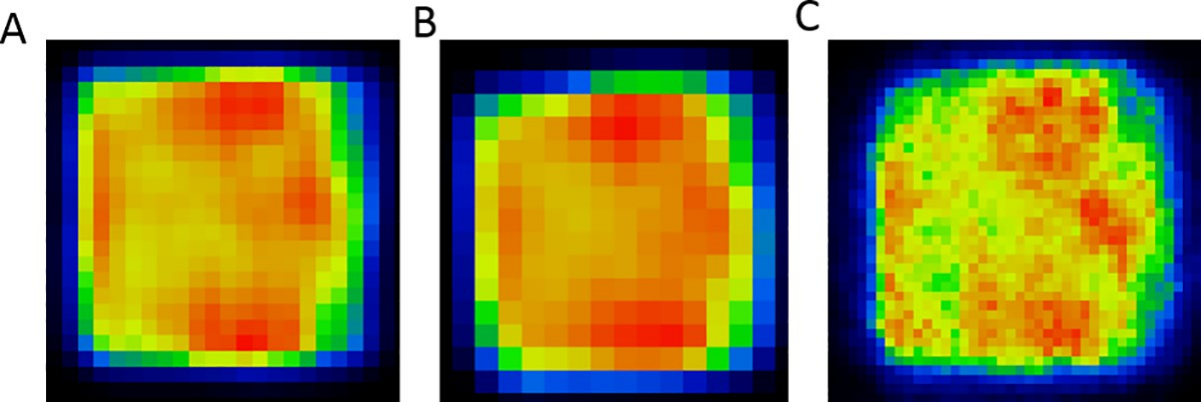
**Representative transaxial slices of the same imitated lesion**: measured and reconstructed by GE Discovery MI PET/CT (A), Mediso AnyScan PET/CT (B), Mediso nanoScan PET/MRI (C).

The simulated 3-D heterogeneous lesion was used to analyze the position dependence of the AnyScan PET/CT. The related representative image slice is shown in Fig 5. Textural parameters and the coefficient of variation of the voxel values within the VOI were calculated from the acquisition of 4 different positions, where the same 3-D patterns was simulated at each location. The reconstructions were performed with and without PSF correction. The calculated values are summarized in Table 3.

**Table 3:** The value of heterogeneity parameters of the same texture acquired in different positions in the field of view.

Distance [mm]	PSF correction	Entropy	Homogeneity	Correlation	Contrast	SRE	LRE	RLNU	LGZE	HGZE	COV [%]	Volume [cm^3^]
**0**	on	6.87	0.29	0.69	80.99	0.63	4.17	0.39	6.73x10^-4^	1830.8	32.4	101.2
	off	6.86	0.27	0.73	65.50	0.63	3.85	0.39	6.46x10^-4^	1875.8	30.4	96.1
**30**	on	6.89	0.28	0.69	79.74	0.63	3.98	0.39	6.6x10^-4^	1909.1	31.6	103.3
	off	6.98	0.26	0.75	70.45	0.65	3.66	0.41	6.66x10^-4^	1899.8	32.8	103.7
**50**	on	6.82	0.29	0.69	73.03	0.64	4.05	0.40	7.16x10^-4^	1720.1	31.8	101.8
	off	6.99	0.26	0.73	79.54	0.64	3.64	0.40	6.49x10^-4^	1953.6	32.8	103.7
**100**	on	6.84	0.29	0.72	68.46	0.66	4.01	0.41	6.42x10^-4^	1882.3	30.2	99.2
	off	6.99	0.27	0.72	80.23	0.64	3.71	0.41	6.34x10^-4^	2000.5	32.0	103.87

### Application 2. Scanner comparison

In the comparison of the texture imaging capability of different scanners, the simulated 3-D heterogeneous lesion was acquired with the GE Discovery MI, the Mediso AnyScan PET/CT and the Mediso nanoScan small animal PET/MRI. For the animal system the step size was reduced from 4 mm to 1 mm. All images were reconstructed with the software provided by the manufacturer. For visual comparison, a representative transaxial slice of the reconstructed images acquired on the three systems are shown in Fig 5.

**Fig 6:**
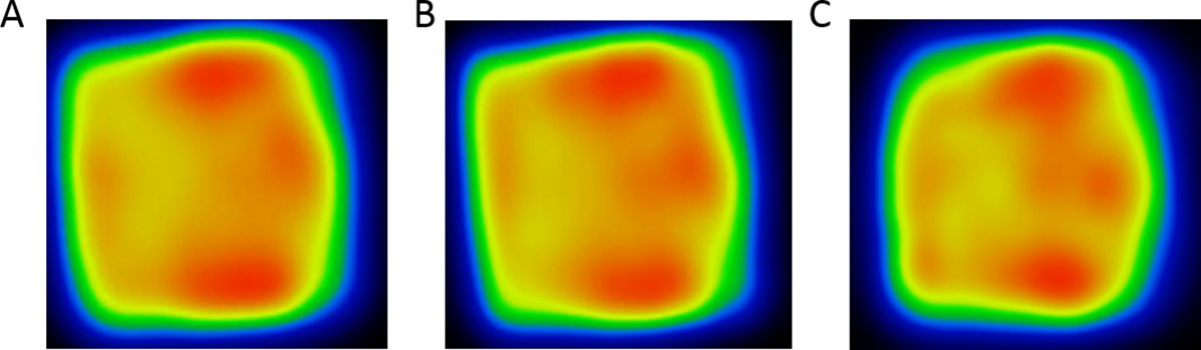
**Representative axial sizes of the lesion measured and reconstructed by different scanners**: Mediso AnyScan PET/CT (panel A), GE Discovery MI (4mm voxel size) on panel B, Mediso nanoScan PET/MRI (1.2mm voxel size) on panel C using spline interpolation.

The same axial slices of the reconstructed images presented on Fig 6, but the voxel sizes were set correspondingly to the spatial resolution of the given scanner (4, 4 and 1.2 mm for the human and the small animal scanners respectively), while the same spline interpolation was applied.

The quantitative comparison of the heterogeneity parameters between scanners are summarized in Tables 4 and 5.

**Table 4:** The values of the heterogeneity parameters of the same pattern measured in three PET scanners.

	Entropy	Homogeneity	Contrast	Correlation	SRE	LRE	RLNU	LGZE	HGZE	COV [%]	Volume [cm^3^]
**Mediso AnyScan PET/CT**	6.72	0.29	68.09	0.68	0.62	4.29	0.38	6.68x10^-4^	1834.3	28.1	101.57
**GE Discovery MI**	6.37	0.35	31.4	0.78	0.5389	5.46	0.32	7.85x10^-4^	1507.1	25.4	96.81
**Mediso nanoScan PET/MRI**	6.66	0.32	31.74	0.85	0.54	4.72	0.33	7.3x10^-4^	1608.0	31.7	1.42

**Table 5:** The values of the heterogeneity parameters of the same pattern measured in three PET scanners after the harmonization of voxel sizes.

	Entropy	Homogeneity	Contrast	Correlation	SRE	LRE	RLNU	LGZE	HGZE	COV [%]	Volume [cm^3^]
**Mediso AnyScan PET/CT**	6.72	0.29	68.09	0,68	0.62	4.29	0.38	6.7x10^-4^	1834.26	28.13	101.57
**GE Discovery MI**	6.89	0.28	78.77	0.67	0.65	3.84	0.41	6.5x10^-4^	1890.78	30.5	101.95
**Mediso nanoScan PET/MRI**	7.3	0.22	126.15	0.63	0.721	3.19	0.48	6.3x10^-4^	1969.73	41.65	1.56

## Discussion

While many plastic phantoms are available for the standardization and evaluation of PET imaging performance and quality assessment with good reproducibility and precise geometrical determination, a method to mimic an arbitrary heterogeneous activity distribution with similar reliability and reproducibility has not yet been developed. Recent attempts include the use of zeolite minerals [8–9], or sources made from alginate [23] as well as various printing technologies [10–13]. These methods still have limitations, either in the complexity in producing the source distribution or having inadequate repeatability and reproducibility properties. In this work we present a novel method which can produce arbitrary irregular shaped distributions of activity concentrations in the field of view of a PET scanner by using a point source that is moved by a precise robotic system. The point source application and the use of a robotic positioning system has been proposed previously although this was used to measure and analyze scanner spatial resolution without continuously moving the source during the acquisition [34–37].

A ^22^Na point source was used in all of our measurements. Although ^22^Na is not used in clinical PET studies, it has several properties that makes it suitable for this particular application and is a good surrogate for clinical PET isotopes such as ^18^F. An accurately calibrated sealed long-lived source as the one used in this work makes the source readily available without the need cumbersome preparation and calibration procedures. The long half-life (2.602 years) allows a distribution to be generated without a significant change in activity during the measurement. The average positron range from a ^22^Na source embedded in an acrylic cube is also very close to 0.66 mm the range of ^18^F positrons in water [36].

The ^22^Na calibration point source was attached to the ensemble of three linear stages and was moved in a 3-D trajectory controlled by Matlab code. Reconstructing the raw data acquired continuously during the motion of the point source resulted in an image of the 3-D activity concentration. The differences of activity concentrations along the trajectory are related to the varied dwell time of the point source. First, using a constant dwell time at each position, the optimization of the step size was performed. Different step size of positioning forms separate dots or connected lines as shown in Fig 2A and 2B. The optimal step sizes have two benefits: 1) the positioning device can generate homogeneous activity distributions in the image; 2) the larger step size holds the potential to create larger volume of the expected distribution during equivalent time. An axial slice of a homogeneous 3-D cube stepped by the selected 4 mm step size is shown in Fig 2C. The coefficient of variation of the voxels within the homogeneous cube is 2.34%, indicating the ability to reliably simulate a homogeneous volume. A set of different geometrical objects like a spherical shell and a heart shape were simulated and reconstructed to highlight the feasibility of creating arbitrary PET image patterns (Fig 3). Nevertheless, the proposed method cannot create real, continuous activity patterns in the FOV but results in inhomogeneous or even homogenous textures on the reconstruction image. Using our method, the calculated numerical values of a given texture index using different PET/CT scanners should be the same and any dissimilarity could give information about the reliability of the actually investigated textural indexes.

In Fig 4 it is demonstrated how the varied dwell time (from 0 to 6 sec) is translated to different activity concentrations (0 to 14 kBq/ml). The reproducibility exceeds what is observed in measurements using conventional phantom which can be attributed to the high precision of the positioning device. The positioning accuracy is 20 μm, the timing accuracy is typically in the range of msec. Moreover, the ^22^Na source is a calibration point source with precisely known activity, geometry and long half-life (2.602 years). Together these individual components and the software control results in a high overall reproducibility. The coefficient of variation of the mean, min, max and the total positioning time from 5 consecutive measurements of a 36 mm diameter hot sphere are summarized in Table 2. The COV for the Max_hot_, Mean_hot_ and Mean_bg_ values were less than 2%, 1% and 4% respectively, suggesting the high reliability of this new method compared to the consecutive study performed by Akamatsu et al. [24] Their findings revealed COV of Max_hot_, Mean_hot_ and Mean_bg_ values between 2.1–3.1, 1.5–2.3 and 9.8–16.5, respectively. The calculated COV of the measurement has to be related to the stochastic nature of acquisition and the reconstruction method, since the reproducibility of the positioning is high.

The sampling frequency across the PET FOV is non-uniform, and the ability to measure and reconstruct a given distribution can therefore be influenced by the radial position of the source. Table 3 summarizes the values extracted from the reconstructed images acquired in 4 different positions with and without PSF correction. The percentage changes caused by the position were generally less than 5%, excluding the Contrast parameter showing 15.5% and 18% with and without PSF correction, respectively.

As we have shown, this method of phantom simulation is capable to produce any irregular shape activity distribution with excellent reproducibility and repeatability. This makes this method an accurate tool to perform comparison between different scanners, especially for texture characterization investigations. As an illustration of this, the same lesion was simulated on two clinical PET/CT systems (GE Discovery MI, and Mediso AnyScan PET/CT) and on a dedicated small animal PET system (Mediso nanoScan PET/MRI). A representative slice of the reconstructed data is shown in Fig 5. The pattern or texture is visually similar in terms of the global intensity distributions for all three images. The smaller voxel size of GE Discovery MI (2.73 mm wide voxels) results in an qualitatively better image quality compared to the Mediso PET/CT (4 mm wide voxels), however, the overall intensity distributions are very similar. In the image acquired on the small animal system (Fig 5C) more texture details appear and more intensity details are visualized. For quantitative comparison, Table 4 presents the corresponding values of radiomics features. Comparing the two clinical scanners, the texture features show greater than 15% differences (except for the Entropy), the Contrast values indicate the highest bias, where the difference is about a factor of 2. After harmonizing the image voxel size, the differences in the textural features from two different clinical systems are reduced to less than 10%, except the Contrast showing a 15% difference (Table 5). Interesting to notice is the application of the spline interpolation (just for visualization, without any manipulation of the voxel values) results in a significantly reduced visual difference between the images acquire on the three scanners (Fig 6). Furthermore, the numerical values of textural indices extracted from the small animal system are very close to the values calculated from phantom data measured in human PET. The easy translation of the phantom size from the human scanner to the small animal system underline the flexibility to scale the distribution of the activity concentration according to the actual PET system. An additional important aspect is that this method also eliminates the so called cold wall effect [38] occurring due to the comparable width of the plastic wall of conventional phantoms to the spatial resolution of small animal systems. It is also essential in the radiomics field to improve the reporting quality and the reproducibility of radiomics studies. Therefore we followed the recommendations available from a recent editorial publication which presented guidelines aiming at the standardization of the image-processing steps before feature extraction and the computation of these features [39].

Beyond well-known multi-device effects on radiomics features in clinical studies, recent publications have investigated these variations in a more refined manners. Multi-device scans were simulated by varying the reconstruction settings and the time per bed positions [28,29]. Furthermore, Orlhac et al. introduced a harmonization method for multicenter radiomics studies in PET [40]. Two different PET/CT systems were used in their study, while a third scanner was simulated by post-filtering one of the acquired data set. Moreover, difficulties arise when considering repeated patient scans because of the physiologic variation in the lesions between scans [41]. Our proposed phantom method is able to overcome the limitations mentioned above, and could support studies involving with multi-device radiomics.

This novel phantom method and the current study includes some limitations. All activity distributions were created in air, while the utilization of additional scattering and attenuating material would result in a more realistic scenario similar to an actual patient scan. Our phantom realization is similar to spatial resolution measurements defined by the NEMA performance protocol, prescribing the activity distribution to be placed in air without any surrounding attenuating and scattering matter. However, phantom simulation methodology could typify radiomics features according to their inter-scanner variability, furthermore can provide data for scanner harmonization process. A textural parameter appearing to be unsuitable according to the protocol in this work without real scattering and attenuating medium will most likely prove to be inadequate according to the more advanced procedure where scatter and attenuation is included. Introducing additional features (scattering and attenuating material, hot background and external activity) may also result in larger deviation between parameter values measured by different scanners and can make the procedure more effective to identify inappropriate indices. Currently we are working to extend our method to better meet to a realistic imaging situation, including adding background activity, attenuating and scattering material and activity elsewhere in and out of the FOV. In addition, the time to simulate a structure is directly proportional to the physical dimension and the complexity of the activity distribution and could potentially be very time consuming.

Recent research in tumor heterogeneity quantification is heavily focused on PET imaging. Under these circumstances, the determination of inter-scanner and inter-vendor differences is one of the most challenging methodological questions. Inter-scanner variations are extremely important in multicenter studies. However, only highly reproducible texture phantom measurements could provide adequate comparison, and to the best of our knowledge there is no appropriate heterogeneity phantom available for this purpose. We also believe that our proposed method could facilitate to develop similar or other kind of texture phantom concepts capable to play a key role in the field of PET radiomics.

## Conclusion

Creating of arbitrary irregular shaped intensity distribution by precise positioning of a point source in the PET scanner field of view is feasible with superior reproducibility. The capability of this concept was demonstrated by simulating a homogeneous cube, a sphere, a spherical shell, a heart shape, and an actual lesion extracted from reconstructed human data. The evaluation of the phantom measurements directly characterizes a PET system allowing to perform comparative studies using different PET scanners.

## References

[1] Aerts HJWL, Velazquez ER, Leijenaar RTH, Parmar C, Grossmann P, Cavalho S, et al Decoding tumour phenotype by noninvasive imaging using a quantitative radiomics approach. Nat Commun. 2014;5 10.1038/ncomms5006 24892406 PMC4059926

[2] Lambin P, Rios-velazquez E, Leijenaar R, Carvalho S, Granton P, Zegers CML, et al Radiomics: Extracting more information from medical images using advanced feature analysis. Proc SPIE—the Int Soc Opt Eng. 2015;73: 389–400. 10.1016/j.ejca.2011.11.036.Radiomics PMC4533986 22257792

[3] Hatt M, Tixier F, Pierce L, Kinahan PE, Le Rest CC, Visvikis D. Characterization of PET/CT images using texture analysis: the past, the present… any future? Eur J Nucl Med Mol Imaging. 2017;44: 151–165. 10.1007/s00259-016-3427-0 27271051 PMC5283691

[4] Cook GJR, Azad G, Owczarczyk K, Siddique M, Goh V. Challenges and Promises of PET Radiomics. Int J Radiat Oncol Biol Phys. The Author(s); 2018; 1–7. 10.1016/j.ijrobp.2017.12.268 29395627 PMC6278749

[5] Hatt M, Tixier F, Visvikis D, Cheze Le Rest C. Radiomics in PET/CT: More Than Meets the Eye? J Nucl Med. 2017;58: 365–366. 10.2967/jnumed.116.184655 27811126

[6] Takeshita T, Morita K, Tsutsui Y, Kidera D, Mikasa S, Maebatake A, et al The influence of respiratory motion on the cumulative SUV-volume histogram and fractal analyses of intratumoral heterogeneity in PET/CT imaging. Ann Nucl Med. Springer Japan; 2016;30: 393–399. 10.1007/s12149-016-1071-1 26955819

[7] Rousset OG, Ma Y, Evans AC. Correction for partial volume effects in PET: principle and validation. J Nucl Med. 1998;39: 904–911. doi: 9591599 9591599

[8] Yip S, McCall K, Aristophanous M, Chen AB, Aerts HJWL, Berbeco R. Comparison of texture features derived from static and respiratory-gated PET images in non-small cell lung cancer. PLoS One. 2014;9: 1–14. 10.1371/journal.pone.0115510 25517987 PMC4269460

[9] Forgacs A, Pall Jonsson H, Dahlbom M, Daver F, Difranco MD, Opposits G, et al A study on the basic criteria for selecting heterogeneity parameters of F18-FDG PET images. PLoS One. 2016;11: 1–14. 10.1371/journal.pone.0164113 27736888 PMC5063296

[10] Barbee DL, Flynn RT, Holden JE, Nickles RJ, Jeraj R. A method for partial volume correction of PET-imaged tumor heterogeneity using expectation maximization with a spatially varying point spread function. Phys Med Biol. 2010;55: 221–236. 10.1088/0031-9155/55/1/013 20009194 PMC2954051

[11] Hatt M, Tixier F, Cheze Le Rest C, Pradier O, Visvikis D. Robustness of intratumour ^18^F-FDG PET uptake heterogeneity quantification for therapy response prediction in oesophageal carcinoma. Eur J Nucl Med Mol Imaging. 2013;40: 1662–71. 10.1007/s00259-013-2486-8 23857457

[12] Galavis PE, Hollensen C, Jallow N, Paliwal B, Jeraj R. Variability of textural features in FDG PET images due to different acquisition modes and reconstruction parameters. Acta Oncol. 2010;49: 1012–6. 10.3109/0284186X.2010.498437 20831489 PMC4091820

[13] Zwanenburg A, Leger S, Vallières M, Löck S, Initiative for the IBS. Image biomarker standardisation initiative. 2016; https://arxiv.org/pdf/1612.07003.pdf

[14] Nyflot MJ, Yang F, Byrd D, Bowen SR, Sandison GA, Kinahan PE. Quantitative radiomics: impact of stochastic effects on textural feature analysis implies the need for standards. J Med Imaging. 2015;2: 41002 10.1117/1.JMI.2.4.041002 26251842 PMC4524811

[15] Buvat I, Orlhac F, Soussan M. Tumor Texture Analysis in PET: Where Do We Stand? J Nucl Med. 2015;56: 1642–1644. 10.2967/jnumed.115.163469 26294296

[16] Makris NE, Huisman MC, Kinahan PE, Lammertsma AA, Boellaard R. Evaluation of strategies towards harmonization of FDG PET/CT studies in multicentre trials: Comparison of scanner validation phantoms and data analysis procedures. Eur J Nucl Med Mol Imaging. 2013;40: 1507–1515. 10.1007/s00259-013-2465-0 23754762 PMC6704482

[17] Soffientini CD, De Bernardi E, Casati R, Baselli G, Zito F. Technical Note: A new zeolite PET phantom to test segmentation algorithms on heterogeneous activity distributions featured with ground-truth contours. Med Phys. 2017;44: 221–226. 10.1002/mp.12014 28066888

[18] Zito F, De Bernardi E, Soffientini C, Canzi C, Casati R, Gerundini P, et al The use of zeolites to generate PET phantoms for the validation of quantification strategies in oncology. Med Phys. 2012;39: 5353–61. 10.1118/1.4736812 22957603

[19] El-Ali H, Ljungberg M, Strand S-E, Palmer J, Malmgren L, Nilsson J. Calibration of a radioactive ink-based stack phantom and its applications in nuclear medicine. Cancer Biother Radiopharm. 2003;18: 201–7. 10.1089/108497803765036364 12804045

[20] Markiewicz PJ, Angelis GI, Kotasidis F, Green M, Lionheart WR, Reader AJ, et al A custom-built PET phantom design for quantitative imaging of printed distributions. Phys Med Biol. 2011;56: N247–N261. 10.1088/0031-9155/56/21/N01 21983701

[21] Berthon B, Marshall C, Holmes R, Spezi E. A novel phantom technique for evaluating the performance of PET auto-segmentation methods in delineating heterogeneous and irregular lesions. EJNMMI Phys. EJNMMI Physics; 2015;2: 13 10.1186/s40658-015-0116-1 26501814 PMC4538718

[22] Miller MA, Hutchins GD. Development of anatomically realistic PET and PET/CT phantoms with rapid prototyping technology. IEEE Nucl Sci Symp Conf Rec. 2007;6: 4252–4257. 10.1109/NSSMIC.2007.4437056

[23] Carles M, Torres-Espallardo I, Alberich-Bayarri A, Olivas C, Bello P, Nestle U, et al Evaluation of PET texture features with heterogeneous phantoms: Complementarity and effect of motion and segmentation method. Phys Med Biol. 2017;62: 652–668. 10.1088/1361-6560/62/2/652 28033121

[24] D’Alessandro B, Madsen M, Samei E, Li X, Wooitan J, Berbaum KS, et al Synthetic positron emission tomography-computed tomography images for use in perceptual studies. Semin Nucl Med. 2011;41: 437–448. 10.1053/j.semnuclmed.2011.06.007 21978446

[25] Papadimitroulas P, Loudos G, Le Maitre A, Hatt M, Tixier F, Efthimiou N, et al Investigation of realistic PET simulations incorporating tumor patient’s specificity using anthropomorphic models: Creation of an oncology database. Med Phys. 2013;40: 1–13. 10.1118/1.4826162 24320465

[26] Orlhac F, Nioche C, Soussan M, Buvat I. Understanding Changes in Tumor Texture Indices in PET: A Comparison Between Visual Assessment and Index Values in Simulated and Patient Data. J Nucl Med. 2017;58: 387–392. 10.2967/jnumed.116.181859 27754906

[27] Castro P, Huerga C, Chamorro P, Garayoa J, Roch M, Pérez L. Characterization and simulation of noise in PET images reconstructed images ଝ. Rev Española Med Nucl e Imagen Mol (English Ed. SEMNIM; 2017; 10.1016/j.remnie.2017.10.025 29678630

[28] Shiri I, Rahmim A, Ghaffarian P, Geramifar P, Abdollahi H, Bitarafan-Rajabi A. The impact of image reconstruction settings on 18F-FDG PET radiomic features: multi-scanner phantom and patient studies. Eur Radiol. European Radiology; 2017;27: 4498–4509. 10.1007/s00330-017-4859-z 28567548

[29] Lucht R, Brix G, Lorenz WJ. Texture analysis of differently reconstructed PET images. Phys Med Biol. 1996;41: 2207–19. Available: https://www.ncbi.nlm.nih.gov/pubmed/8912391 8912391 10.1088/0031-9155/41/10/025

[30] Hsu DFC, Ilan E, Peterson WT, Uribe J, Lubberink M, Levin CS. Studies of a Next-Generation Silicon-Photomultiplier–Based Time-of-Flight PET/CT System. J Nucl Med. 2017;58: 1511–1518. 10.2967/jnumed.117.189514 28450566

[31] Nagy K, Toth M, Major P, Patay G, Egri G, Haggkvist J, et al Performance Evaluation of the Small-Animal nanoScan PET/MRI System. J Nucl Med. 2013;54: 1825–1832. 10.2967/jnumed.112.119065 23990683

[32] Tixier F, Le Rest CC, Hatt M, Albarghach N, Pradier O, Metges J-P, et al Intratumor Heterogeneity Characterized by Textural Features on Baseline 18F-FDG PET Images Predicts Response to Concomitant Radiochemotherapy in Esophageal Cancer. J Nucl Med. 2011;52: 369–378. 10.2967/jnumed.110.082404 21321270 PMC3789272

[33] Koopman D, van Osch JAC, Jager PL, Tenbergen CJA, Knollema S, Slump CH, et al Technical note: how to determine the FDG activity for tumour PET imaging that satisfies European guidelines. EJNMMI Phys. EJNMMI Physics; 2016;3 10.1186/s40658-016-0158-z 27682837 PMC5040656

[34] Liinssjukhus NA. Point-Source Scanner for Assessment of Gamma Camera Performance. 1992;20.

[35] Panin VY, Kehren F, Michel C, Casey M. Fully 3-D PET reconstruction with system matrix derived from point source measurements. IEEE Trans Med Imaging. 2006;25: 907–921. 10.1109/TMI.2006.876171 16827491

[36] Alessio AM, Stearns CW, Tong S, Ross SG, Kohlmyer S, Ganin A, et al Application and evaluation of a measured spatially variant system model for PET image reconstruction. IEEE Trans Med Imaging. 2010;29: 938–949. 10.1109/TMI.2010.2040188 20199927 PMC2903538

[37] Murata T, Miwa K, Miyaji N, Wagatsuma K, Hasegawa T, Oda K. Evaluation of spatial dependence of point spread function-based PET reconstruction using a traceable point-like 22 Na source. EJNMMI Phys. EJNMMI Physics; 2016; 0–9. 10.1186/s40658-016-0162-3 27783373 PMC5080272

[38] Lajtos I, Czernin J, Dahlbom M, Daver F, Emri M, Farshchi-Heydari S, et al Cold wall effect eliminating method to determine the contrast recovery coefficient for small animal PET scanners using the NEMA NU-4 image quality phantom. Phys Med Biol. 2014;59: 2727–46. 10.1088/0031-9155/59/11/2727 24800813

[39] Vallieres M, Zwanenburg A, Badic B, Cheze-Le Rest C, Visvikis D, Hatt M. Responsible Radiomics Research for Faster Clinical Translation. J Nucl Med. 2017; jnumed.117.200501. 10.2967/jnumed.117.200501 29175982 PMC5807530

[40] Orlhac F, Boughdad S, Philippe C, Stalla-Bourdillon H, Nioche C, Champion L, et al A post-reconstruction harmonization method for multicenter radiomic studies in PET. J Nucl Med. 2018;2: jnumed.117.199935. 10.2967/jnumed.117.199935 29301932

[41] Tixier F, Hatt M, Cheze C, Rest L, Pogam A Le, Corcos L, et al Reproducibility of Tumor Uptake Heterogeneity. J Nucl Med.: 693–701. 10.2967/jnumed.111.099127 22454484 PMC3779464

